# The effectiveness of new urban trail infrastructure on physical activity and active transportation: a systematic review and meta-analysis of natural experiments

**DOI:** 10.1186/s12966-025-01729-4

**Published:** 2025-03-27

**Authors:** Isaak Fast, Christie Nashed, Jack Lotscher, Nicole Askin, Hannah Steiman De Visser, Jonathan McGavock

**Affiliations:** 1 Diabetes Research Envisioned and Accomplished in Manitoba (DREAM) Research Theme, Children’s Hospital Research Institute of Manitoba, Winnipeg, Canada; 2 Faculty of Kinesiology, University of Manitoba, Winnipeg, Canada; 3 School of Medical Sciences, University of Manchester, Manchester, UK; 4 School of Human Kinetics, Faculty of Health Sciences, University of Ottawa, Ottawa, Canada; 5 Neil John MacLean Library, Rady Faculty of Health Sciences, University of Manitoba, Winnipeg, Canada; 6 College of Medicine, Rady Faculty of Health Sciences, University of Manitoba, Winnipeg, Canada; 7 Department of Pediatrics and Child Health, Rady Faculty of Health Sciences, University of Manitoba, Winnipeg, Canada

**Keywords:** Cycling, Built environment, Exercise, Active transportation, Natural experiments, Meta analysis

## Abstract

**Background:**

Cities in Western countries are investing billions of dollars in new cycling infrastructure (urban trails) to support active transportation (AT) and leisure-type physical activity (PA). Little empirical evidence exists on the effectiveness of urban trails on changes in AT or PA.

**Design and methods:**

We searched CINAHL, OVID, SPORTDiscus, Transport Research International Documentation (TRID), Web of Science Core Collection and Google Scholar for articles published from 2010 to 2023. We included controlled experimental studies that reported PA, AT or trail counts as outcome measures before and after construction of an urban trail. A modified risk of bias tool was employed to assess the methodological quality of each selected study (Prospero ID: CRD42023438891).

**Results:**

Three independent reviewers screened abstracts from 3936 articles identified in the original search and identified 24 articles that met inclusion criteria: 11 studies (*n* = 11,464) that measured changes in PA, 8 studies (*n* = 92,001) that measured changes in cycling traffic and 5 studies (*n* = 4,958,203) that measured changes in rates of AT/cycling. Meta-analysis revealed that new trails increased PA levels among individuals in proximity to one, compared to those living in control areas (SMD = 0.12; 95% CI: 0.04, 0.20; I^2^ = 73%; *n* = 11,464). This effect was marginally stronger when data were restricted to individuals living in closest proximity to trails (SMD = 0.14; 96% CI: 0.06 to 0.25, I^2^ = 74%; *n* = 8234). Meta-analyses were not possible for measures of AT and cycling counts. All studies were at high risk of bias due to a failure to adhere to reporting guidelines for quasi-experimental studies.

**Conclusions:**

There is limited but intriguing evidence that the addition of protected urban trails increases daily PA for individuals living in neighbourhoods that receive them. The strength of this evidence could be enhanced with the application of and adherence to principles of causal inference and increased diversity of individuals included in study designs.

**Supplementary Information:**

The online version contains supplementary material available at 10.1186/s12966-025-01729-4.

## Introduction

Implementing protected infrastructure to support cycling or walking (referred to herein as urban trails) is the most common governmental strategy for providing citizens with safe spaces for active transportation (AT) and physical activity (PA) [1–3]. Although the expansion of urban trails is the fastest-growing municipal strategy for reshaping the urban built environment [4], there is little empirical evidence on whether they increase AT or PA in urban populations.

Randomized controlled trials are impractical or not feasible to study the health impacts of changes to the built environment. As such, well controlled natural experiments offer the best empirical design to estimate causal effects of adding new urban trails on individual health outcomes or behaviours, including PA and AT [5, 6]. To date, several systematic reviews have tried to summarize the available evidence for the effectiveness of adding trails to urban environments on individual-level PA and walking/cycling behaviour for citizens living adjacent to them [7–10]. These reviews suggest that the implementation of urban trails leads to an increase in PA levels for individuals living in proximity to them. The causal nature of this evidence is limited however as some failed to include natural experiments that were published in transportation journals [9], others included natural experiments without control conditions and observational studies [7, 8] and some reviews did not meta-analyze results across multiple studies [7, 8]. Although journals have begun to adopt and adhere to guidelines for reporting natural experiments [11, 12] and scientists have called for more robust study designs that meet assumptions for causal inference [10], to the best of our knowledge no systematic review of urban trail natural experiments to date has adopted the most recent reporting guidelines for natural experiments when assessing the risk of bias for previously published studies [11, 13]. A more precise estimate of the effect of urban trails on both PA and AT and the overall quality of this evidence is needed to inform policy makers and expert guidelines for the promotion of PA through changes to the built environment [14–17].

The purpose of this systematic review and meta-analysis was to overcome the limitations of previous systematic reviews and provide a comprehensive estimate of the causal effects of implementing new urban trails on individual changes in PA and AT behaviours. We treated PA and AT as separate outcomes as urban trails can lead to increased leisure time PA alone, AT behaviours alone or both [18].

## Research design and methods

### Data sources, search strategy, and eligibility criteria

This review is reported according to the PRISMA guidelines [19]. An initial search strategy was developed and received a PRESS from another information specialist [20]. We then searched CINAHL with Full Text (EBSCO), EMBASE (Ovid), MEDLINE (Ovid), SPORTDiscus (EBSCO), Transport Research International Documentation (TRID), Web of Science Core Collection (Clarivate), and Google Scholar for grey literature. The systematic search of these databases was conducted to identify studies of natural experiments with a valid pre/post design and control condition published between January 1, 2010, and July 21, 2023. The year 2010 was chosen as the cut-off year as it serves as a salient time point when many governments began investing more significantly in creating cycling infrastructure and preliminary search efforts suggested most studies of natural experiments began to be published around 2010. The protocol was registered on PROSPERO (CRD42023438891). Institutional ethics approval was not required for this systematic review and meta-analysis as individual level data were not used for these analyses.

### Classification of the intervention

Natural experiments of either high-comfort (cycle tracks, local street bikeways, bike paths) or medium-comfort (multi-use paths) trails according to the CAN-BICS Classification System [21, 22] were included in the review. Specifically, natural experiments of urban trails had to focus on the impact of high or medium comfort trails that were separated from the roadway via physical distance or a concrete barrier.

### Inclusion criteria

We included studies that met the following inclusion criteria: (1) natural experiments of a new urban with a control arm/control area; (2) urban trails had to be cycling infrastructure that was protected from vehicular traffic; and (3) experiments had to report changes in either daily/weekly physical activity, rates of active transportation or cycling counts as a main outcome measure. We excluded (1) studies that did not have a control group; (2) studies that assessed a new trail that was not protected from vehicle traffic (i.e. low comfort trail) as the intervention, (3) studies that did not have a measure of PA, rates of AT/cycling or cycling or pedestrian traffic and (4) studies that were not published in English.

### Outcomes of interest

The primary outcome of interest was daily PA, reported as minutes per day or week of physical activity, moderate to vigorous intensity PA or MET-mins per day or week. Secondary outcomes included rates of AT and cycling/pedestrian traffic along the trail.

### Study selection

**Fig. 1:**
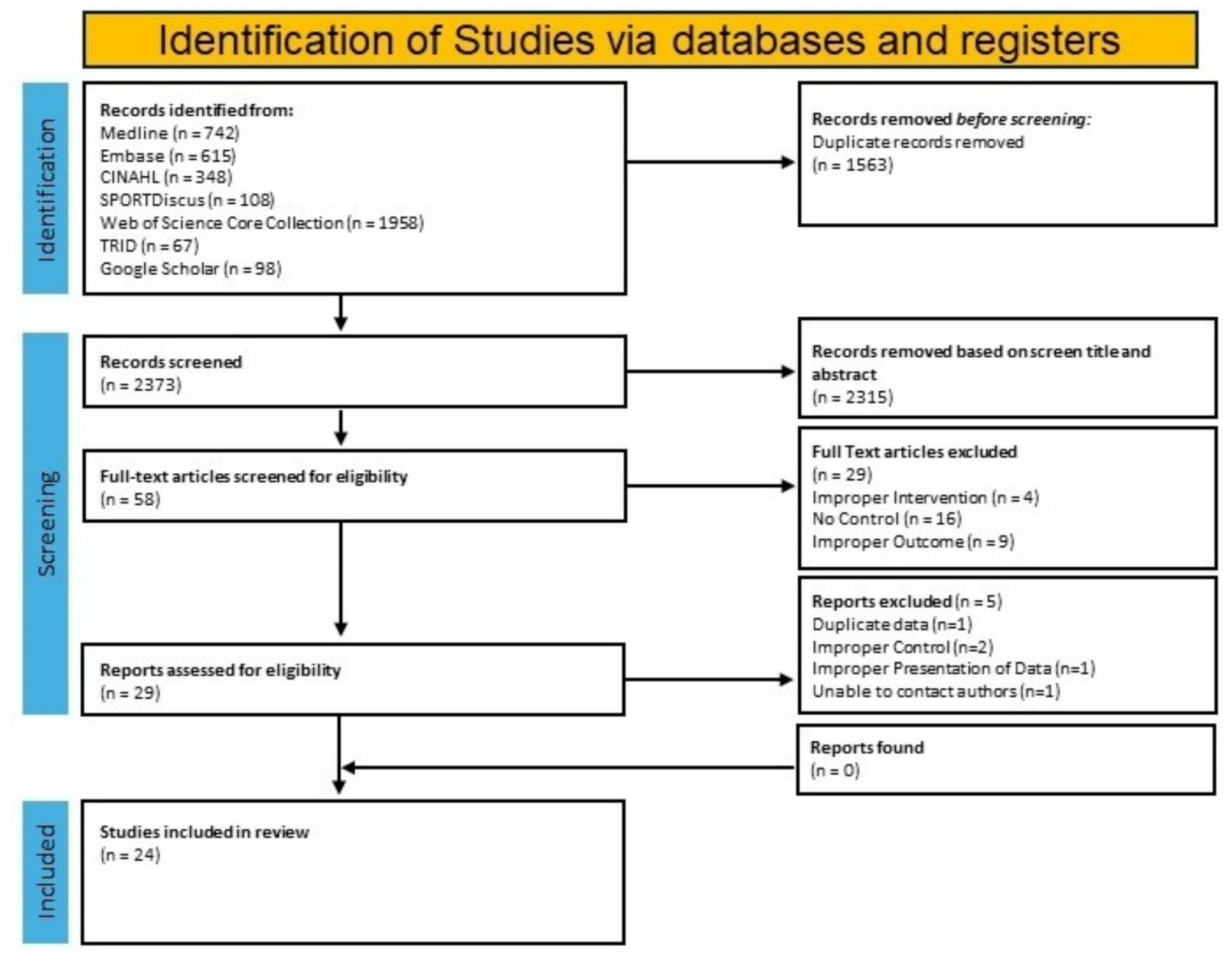
Flow chart of systematic selection of studies

The systematic search yielded 3,936 abstracts (Table 1 appendix for results of the search strategy) that were screened independently by four reviewers (HSD, CN, IF, JL) to determine eligibility. Prior to the abstract screening, a preliminary screening of the first 30 articles yielded an agreement rate between the four reviewers of 100%. The same reviewers screened the remaining titles and abstracts, with conflicts being resolved collaboratively under the guidance of an independent reviewer (JMc). A flow chart of study selection from search to meta-analysis is provided in Fig. 1. Of the 3936 articles identified, 1583 were duplicates, and 2315 did not meet inclusion for full-text review. Of the 58 articles that were full-text screened for eligibility, four did not report an urban trail that met inclusion criteria, 16 did not have a control group, and nine did not report data for the outcomes of interest. A further five were excluded after passing the initial stages as they were either duplicate publications, did not present data correctly, improperly identified a control group or did not provide sufficient data for interpretation (i.e. reported Cohen’s D values only).

**Table 1:** Study characteristics for natural experiments with controlled per-post designs that Met inclusion criteria

Study	Country	Intervention/ control (*n*)	Trail Type	Population (age-yrs)	Method to measure outcome	Trail Distance (kms)	Buffer Distance	Follow- up Time
**Studies that assessed Physical Activity**								
Aldred et al. 2019	UK	750/962	MUP	Adults	IPAQ	11.27	500 m	12 mos
Aldred et al. 2021	UK	750/962	MUP	Adults	IPAQ	11.27	2 km	5 yrs
Crane et al. 2017	Australia	448/398	PBL	Adults	Survey	2.4	3 km	2 yrs
Frank et al. 2019	Canada	239/285	MUP	46.2	Survey	2	1 km	2 yrs
He et al. 2021	China	960/280	MUP	49.4	Survey	102	5 km	3 yrs
He et al. 2022	China	766/254	MUP	50.1	Survey	102	5 km	3 yrs
Hunter 2021	Ireland	2005/414	MUP	50.3	GPAQ	30	1.6 km	6 yrs
Pazin et al. 2016	Brazil	380/329	MUP	Adults	Survey	2.3	1.5 km	3 yrs
Stappers et al. 2021	Netherlands	442/400	MUP	57.8	Accelerometer	2.3	N/A	12 mos
West et al. 2011	USA	95/74	MUP	Adults	Survey	8	1.6 km	12 mos
West et al. 2015	USA	118/85	MUP	Adults	Survey	3.01	1.6 km	2 yrs
**Studies that assessed Active Transportation**								
Aldred et al. 2021	UK	750/962	MUP	Adults	Survey	11.27	500 m	12 mos
Keall et al. 2015	New Zealand	556/234	MUP/PBL	N/A	Survey	N/A	N/A	12 mos
Frank et al. 2021	Canada	239/285	MUP	46.2	Survey	2	300 m	2 yrs
Brown et al. 2016	USA	536/910	“High-Comfort”	41.7	Survey	N/A	400 m	12 mos
Goodman et al. 2013	UK	2,751,198/2,178,498	MUP/PBL	N/A	Census	N/A	1.6 km	10 yrs
Patterson et al. 2023	UK	6373/19,374	PBL	Adults	Census	360	N/A	10 yrs
**Studies that assessed Cycling Traffic**								
Auchincloss et al. 2019	USA	21,488/18,746	MUP	N/A	Automated Counts	2.5	7.5 km	3 yrs
Fitzhugh et al. 2010	USA	N/A	MUP	30.0	Manual Counts	4.64	N/A	2 yrs
Garber et al. 2022	USA	N/A	MUP/PBL	36.0	Eco-Counter	22.41	1.6 km	2 yrs
Hans et al. 2017	Denmark	50,954	MUP	N/A	Eco-Counter	18	N/A	2 yrs
Heesch et al. 2016	Australia	169/132	MUP/PBL	N/A	Manual Counts	17	N/A	4 yrs
Nguyen et al. 2015	Singapore	N/A	MUP	N/A	Manual Counts	11.3	N/A	2 yrs
Rissel et al. 2015	Australia	240/272	MUP	Adults	Manual Counts	2.4	2.5 km	12 mos
Xiao et al. 2022	France	N/A	MUP	N/A	Eco-Counter	16	2 km	6 yrs

IPAQ = international physical activity questionnaire; GPAQ = Global physical activity questionnaire; MUP = multi use path; PBL = protected bicycle lane; N/A = not available; km = kilometers; yrs = years

### Data extraction

Data was extracted by one reviewer (IF) and confirmed by a second (JL). The data extracted included demographic characteristics of participants (measures of socio-economic status, age, education level, gender and race), the intervention (buffer distance that separated individuals into intervention or control conditions, length of the trail and trail type), timing of follow up measures and the outcomes of interest data. Studies varied in PA reporting methods, having both subjective and objective measures. Data extracted from self-reported PA and objective measures were extracted as they were reported in each manuscript. Corresponding authors were contacted for any missing data. Depending on the necessity of the missing information, if data were unable to be retrieved the study or data were excluded from the meta-analysis.

### Risk of bias assessment

We used a modified risk of bias tool to rate the quality of the studies using the TREND reporting guidelines as a framework [11, 12]. The tool we created was categorized into 3 broad areas of potential sources of bias (study design, data analysis and report and reporting of intervention and participant characteristics). Key reporting metrics from TREND reporting guidelines, core elements of urban trail natural experiments (type of trail, length, surface type, buffer distance to categorize intervention and control areas) as well as key aspects of reporting PA-related outcomes (time of year, objective/subjective, adjusting for confounding) were used to assess the overall risk of bias. These varying metrics were reported on a binary scale with 1 for an element that was reported and 0 when it was not reported. Scores were summed and each study assigned a score out of a maximum of 35.

### Statistical analyses

A DerSimonian and Laird random-effects model was used to compare standardized mean differences with 95% confidence intervals between changes in PA, treated as a continuous outcome between individuals living in areas that lived within the buffer area defined as receiving an urban trail (intervention) compared to individuals living in areas outside the buffer area (control). An additional subgroup analysis was performed to determine if the effect size was different for studies that reported a dose-response effect of the trail related to the proximity of individuals to the new trails. We planned to conduct a Mantel-Haenszel random effects odds ratio analysis to test for differences in rates of AT/cycling trips between individuals in intervention and control areas, however all but one study reported pooled, adjusted effect sizes without raw data for intervention and control areas before and after the construction of a new trail. Data were analyzed using Revman 5.4 (Cochrane Collaborative).

## Results

After systemic searches of each database, 3,936 citations were included in a preliminary screen, 1,563 articles were deemed duplicates, leaving 2,373 articles to be screened (Table 1 appendix). At the end of the title and abstract screening, 2,238 papers were excluded, there was a 94.3% agreement rate between the four reviewers for the preliminary title and abstract screen and 135 were in conflict among four reviewers (IF, HSD, CN, JL) (Fig. 1). The 135 papers with conflicting decisions were resolved by the four screeners and one investigator (JMc) to achieve consensus for eligibility for full-text screening. After all conflicts were resolved, 58 papers were included in the full-text screen where 29 were deemed eligible for data extraction. Once the data extraction process began, 5 studies [23–27] were deemed ineligible, as they were either duplicates or did not meet inclusion criteria, either through the presentation of data, inability to contact authors for missing data, or missing control groups. This led to a final sample of 24 studies that met the inclusion criteria [28–51]. Of the 24 natural experiments studied, 11 measured PA as the primary outcome [28–38], 8 measured changes in cycling traffic [39–46], 5 measured the changes in AT [47–51] and 1 reported changes in both PA and AT [27].

Among the 11 studies that assessed PA [28–38] (*n* = 11,464) the proportion of females was 55% in the intervention arm, and 59% in the control arm. The median age was 50 years in the intervention arm (range: 10, 85), and 53 years (range: 10, 85) in the control arm. Six [28, 29, 31, 35, 37, 38] of the 24 studies reported on the ethnicity of the participants and within these studies > 75% of participants were white. For studies that assessed AT or cycling counts (*n* = 13)^39–51^ the proportion of females in the intervention arm was 49% with a mean age of 36 years (range: 10–85 years), and in the control arm, the female proportion was 47%, with a mean age of 38 years (range: 10–85 years). Only one study included outcome data for children and adolescents [47].

Intervention characteristics of the studies included in the analyses are provided in Table 1. In total, the 24 studies examined the construction of 439 km of new protected urban trails, with a median length of 41 km of new trail (range 2-102 kms). Within the 11 studies that measured changes in PA [28–38], the mean control group sample size was 269, with a mean intervention sample size of 329. The mean follow-up period was 3.1 years (range: 1 to 5 years). Among the 11 studies that assessed PA, only 1 used an objective measure [36]. Among the 8 studies that quantified changes in cycling traffic, 4 used objective measures (Eco-Counters, Montreal, Qc) [39, 41, 42, 45] and 4 used field observations [40, 43, 44, 46]. Among the 6 studies of AT/cycling frequency, 2 used census data [50, 51] and 4 used field surveys [27, 47–49] to measure outcomes. One study reported both PA and AT, using a self-reported survey.

### Effectiveness of new trails on physical activity levels and cycling traffic

**Fig. 2:**
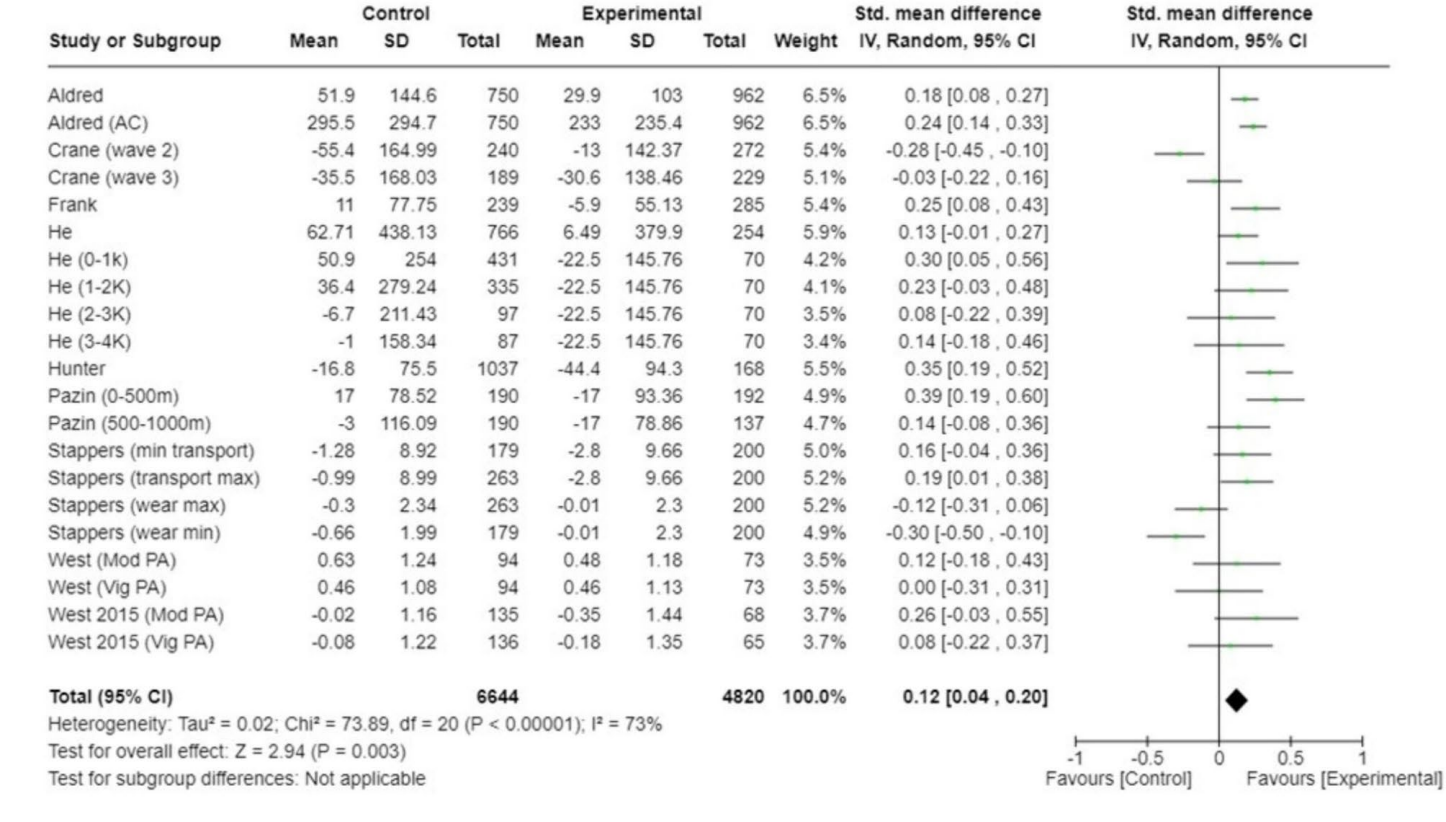
Meta-analysis of 11 studies that reported changes in minutes of physical activity following implementation ofa new urban trail

Among the 11 studies that provided 21 comparisons [28–38], PA increased in individuals who were exposed to the new trail compared to those not exposed [SMD = 0.12; 95% CI: 0.04, 0.20; I^2^ = 73%; *n* = 11,464] (Fig. 2). Restricting the analysis to individuals in closest proximity to the trail (*n* = 10 studies; 14 comparisons) modestly increased the effect size but reduced the precision of the estimate for adding an urban trail on individual PA [SMD = 0.14; 96% CI: 0.06 to 0.25, I2 = 74%; *n* = 8234; Figure s1 Appendix)]. A total of six studies [27, 47–51] provided 9 effect estimates with confidence intervals for the change in AT/cycling following the construction of a new trail (Table 2). Of the 9 comparisons, the mean odds ratio was 1.41 (range 1.01–3.52) and 4 comparisons from 4 studies [45, 48, 50, 51] found a positive effect of adding a new trail on rates of AT/cycling, and 5 comparisons from 3 studies [29, 49, 51] had confidence intervals that included the null. A total of eight studies [39–46] provided 10 comparisons for the effect of a new trail on changes in cycling traffic along intervention and control trails. Six comparisons from 5 studies [39–41, 43, 45] reported effect sizes from group by time interactions, while 4 comparisons from 3 studies [42, 44, 46] only reported pre-post effect sizes for intervention areas (Table 3). Three of six comparisons that reported a group x time interaction reported a significant increase in cycling counts. All four comparisons that only reported pre-post data, reported an increase in cycling counts.

**Table 2:** Summary of studies examining effectiveness of urban trails on changes in active transportation

Study	Trail type	Method	Outcome	Int	Control	OR	95% CI
Aldred 2021 W1	MUP	Survey	Past week AT	770	962	1.03	0.99–1.07
Aldred 2021 W2	MUP	Survey	Past week AT	708	902	1.02	0.98–1.05
Aldred 2021 W3	MUP	Survey	Past week AT	668	830	1.01	0.98–1.05
Keall et al. 2015	MUP/PBL	Survey	Past week AT	490	202	1.37	1.08–1.73
Frank et al. 2021	MUP	Survey	Last 2 days cycling trips	239	285	3.52	1.54–8.03
Brown et al. 2016	“High-Comfort”	GPS/ Accelerometer	Active trips	NR	NR	UInt	UInt
Goodman et al. 2013	MUP/PBL	Census	Cycling to work	2.75 M	2.18 M	1.09	1.07–1.11
Patterson et al. 2023 (cycle)	PBL	Census	Cycle Commute	6,373	19,373	1.08	0.92–1.26*
Patterson et al. 2023 (walk)	PBL	Census	Walking Commute	6,373	19,374	1.18	1.06–1.32

MUP = multi use path; PBL = protected bicycle lane; NR = not reported; OR = odds ratio; CI = confidence intervals

*=Gender stratified analyses yielded AOR = 1.56; 95% CI 1.16 to 2.10 for women and

AOR = 0.91; 95% CI 0.76 to 1.10 for men. Uint = odds ratios provided without confidence intervals and were uninterpretable relative to other studies reporting odds ratios for changes in rates of AT following urban trail implementation

**Table 3:** Effectiveness of urban trails on changes in cycling traffic

Study	Intervention type	Design	Outcome units	Intervention Baseline	Intervention Post	Effect Size (confidence intervals)	Group x Time Interaction
Auchincloss et al. 2019	MUP	Pre-Post Control	Persons / hour	100 ± 45	116 ± 48	+ 5% (+ 4, + 9%)	Yes
Fitzhugh et al. 2010	MUP	Pre-Post control	Persons / 2 h	4.5 (2.5-6)	13 (11–15)	*p* = 0.001	Yes
Garber et al. 2022	MUP/PBL	Synthetic Control	Miles ridden/month	NR	NR	+ 1922 (-394, + 3542)	Yes
Hans et al. 2017	MUP	Pre-Post Control	Cyclists / hour	126 (122,130)	206 (195,210)	+ 61 vs. + 7%	No
Heesch et al. 2016	MUP/PBL	Pre-Post Control	Cyclists / month	---	---	+ 225 (+ 78, + 372)	Yes
Nguyen et al. 2015	MUP (wide)	Pre-Post	Cyclists / hour	45 ± 24.5	57.6 ± 34.9	+ 28%	No
Nguyen et al. 2015	MUP (small)	Pre-Post	Cyclists / hour	38.8 ± 32.4	55.8 ± 36.6	+ 44%	No
Rissel et al. 2015	MUP	Pre-Post	Cyclists/Month	201(B) & 812(A)*	395 (B) & 1001 (A)*	+ 97% (B) & +23% (A)	No
Xiao et al. 2022	MUP (Paris)	Interrupted time series	Cyclists / day	2009 ± 1507	2703 ± 2351	218 (-189, 626)	Yes
Xiao et al. 2022	MUP (Lyon)	Interrupted time series	Cyclists / day	1336 ± 1120	1663 ± 1223	34 (-65, 133)	Yes

MUP = multi use path; PBL = protected bicycle lane; NR = not reported

* = data were collected at two sites along the new trail (A and B). Pre-post intervention counts are provided for both sites

### Risk of bias

The overall quality of each study was evaluated on 29 different metrics (Table S2 of the appendix). Out of a maximal score of 35 for each study and the average score was 24 (range 15–28). Of the studies included in the data extraction, 0 reported intention to treat analyses, 1 of 24 studies [31] reported whether incentives were used as a form of recruitment aid, 1 study used an objective measure of PA [36], 5 of 24^27,28,41,45,50^ provided a power calculation, 3 of 24^36,40,44^ reported the surface of the new trail, and 7 of 24^27,32,39,43–45^ reported the preceding geography of the area. Most studies reported sources of confounding and strategies to overcome confounding bias (16/24 and 22/24, respectively). Only 12 out of 24 studies reported race for individuals surveyed, and none of those provided results disaggregated by race or sex.

## Discussion

To the best of our knowledge, this is the most comprehensive systematic review and meta-analysis to date of the effects of natural experiments of urban trails on changes in individual PA levels, rates of AT and cycling traffic. The main finding of the meta-analysis was that there is a small but significant increase in PA among individuals living close to a new trail following trail implementation. These improvements in PA appear to be coupled with increased rates of AT and cycling traffic; however, data availability limited statistical comparisons. Lastly, we identified significant gaps that could be addressed in future studies including improvements in methodological design, particularly strategies to enhance causal inference and the use of objective measures to assess PA and AT outcomes. Additionally, the external validity of these studies is low due to a lack of data available for various segments of the population, particularly children and youth and individuals from structurally oppressed groups.

The expansion of cycling infrastructure is the fastest growing and most expansive change to the built environment that supports PA in cities in Asia, Europe and North America. Several research groups in Europe, China, Canada and the US have capitalized on these natural experiments to determine if they nudge citizens to engage in more PA or AT [28–51]. Two previous systematic reviews examined the pooled effects of these interventions on measures of PA for individuals living in proximity to them [7, 9]. Both studies suggested a positive effect of new trails on PA however either failed to meta-analyse results as they included observational studies without a control condition or failed to include studies of controlled natural experiments [7]. The other meta-analysis failed to include natural experiments published in transportation journals [9]. The current meta-analysis expands on these two studies with the addition of 5 natural experiments and supports previous conclusions. Specifically, with a nearly three-fold larger sample size (*n* = 11,343 vs. 4,081), we found an almost identical positive 12% increase in PA following the implementation of new urban trails [9]. Sub-group analyses suggest this effect is modestly higher for individuals living closer to the trail than for individuals further away. Additionally, we found that only 4 of 9 comparisons (6 studies) reported positive effects of implementing new trails on rates of AT or cycling frequency while 7 of 10 comparisons (8 studies) reported increased rates of cycling traffic following construction of a new trail. Collectively, these data provide evidence that the addition of protected spaces for cycling and walking, in the form of urban trails, is associated with modest increases in PA, AT and potentially cycling traffic for individuals living in areas 500 m-1 km from the trail.

Natural experiments provide the most rigorous methodological design to determine the causal effects of an intervention that is not suitable for a randomized control trial [6]. The credibility of the evidence emerging from a natural experiment is however dependent on the quality of the methods used to study it [52, 53]. Previous systematic reviews have identified the high risk of bias evident in the evaluations of natural experiments of urban trails [7–10]. These include (1) the risk of confounding bias if individuals in control and intervention areas are dissimilar at baseline (exchangeability assumption); (2) the risk of selection bias if all individuals do not have an equal chance of receiving the intervention (positivity assumption) and (3) the risk of spillover effects if additional interventions (simultaneous urban cycling policies) or unintended consequences of urban trail interventions (gentrification of neighbourhoods) occur in conjunction with the implementation of a new trail (consistency assumption) [54]. The majority of studies of natural experiments to date have failed to properly address these assumptions through design approaches. For example, few studies ensured baseline outcome data were similar prior to the intervention or matched control neighbourhoods with intervention neighbourhoods on baseline demographic information, or even reported demographic information for control arms [26, 35, 39, 41, 43, 44, 46, 47, 50]. In the absence of information on recruitment, it was unclear if individuals recruited were equally likely to use the trail in intervention and control areas. Lastly, few studies captured PA outcomes using valid tools, during the same season in the pre and post time periods [26, 35, 45] Accordingly, the risk of bias for these natural experiments is high and researchers should draw from the growing body of literature on the use of difference-in-difference designs [13, 52, 55] or interrupted time series with synthetic controls when designing the evaluation of future natural experiments of urban trails to more rigorously estimate causal effects of urban trails on health or behavioural outcomes.

Urban trails are not equally distributed within cities. Studies in Canada and the US reveal that investments in protected spaces for cycling and walking are often made in affluent areas with fewer individuals from racialized groups [56, 57]. Additionally, individuals from structurally oppressed groups (women, newcomers, racialized groups) are less likely to use existing urban trails [55]. The results from this systematic review add to this body of knowledge as few experimental studies recruited or document the impact of urban trails on individuals from structurally oppressed groups or other segments of the urban population, particularly children and adolescents. Exclusion of racially oppressed populations have several possible effects on the summary results presented here [58]. First, they could inflate the effect size if urban trails only meet the needs of certain racial groups [59]. Exclusion of oppressed groups could also diminish the effect size if individuals from racialized groups in some cities are more likely to rely on forms of active transportation (i.e. urban Indigenous populations in Canada). Overall however, the lack of data on the effectiveness of urban trails for increasing PA or AT among structurally oppressed limits the external validity of these findings and transferability to all urban populations [58, 59]. As cities implement policies for racial, gender and sexual equity, investigators studying natural experiments should make efforts to include diverse samples of individuals in their study designs and embrace equity and justice-informed race-based data collection [60].

The data presented here provide some insight into the design of studies focused on urban trail natural experiments. In brief, future studies should consciously consider measuring and reporting key variables outlined in the TREND statement reporting guidelines [11, 12], key assumptions for difference-in-difference designs [13], and best practices for collecting objectively measured PA [61]. With respect to the study of urban trails, future studies should clearly delineate the intervention and control areas using specific buffer areas, provide detailed descriptions of the urban trail being implemented (duration, type, width) and collect, report and balance individual- and area-level variables that could confound comparisons between intervention and control areas. Finally, researchers are encouraged to collect data from a diverse group of individuals, particularly structurally or racially oppressed populations that are often excluded from this type of research.

### Strengths and limitations

To our knowledge, this is the most extensive systematic review and meta-analysis of controlled studies of natural experiments of new protected cycling/walking paths to date. The study was strengthened by the broad search strategy used to date and pre-registered methods. Despite these strengths, there are still several limitations of this review, particularly the lack of data available for meta-analysis within most published studies examining changes in rates of AT/cycling trips. Additionally, we restricted analyses to scientific papers written in English potentially influencing the effect estimates. Furthermore, there were a relatively small number of natural experiments available in the literature and most had limited sample sizes, limiting the generalizability and precision of our estimates. The risk of confounding and selection bias is high do a failure to collect and adjust for neighbourhood-level confounding. For example, most studies did not match or control for differences in demographic (age, gender, race) or environmental factors (trail connectivity, parks, fitness centres, socio-economic status) for individuals living within intervention and control areas. For example, nine studies did not provide sufficient information for participants at baseline, therefore it is unclear how comparable populations were prior to the implementation of new trail. Differences in age, activity level or health status could contribute to positive effects seen with trail implementation. The wide variation in reporting methods was also a limitation as not all the data could be pooled for meta-analysis. Few studies addressed the risk of spillover effects of gentrification of a neighbourhood following the construction of a new trail (part of the stable unit treatment value assumption [62]). Finally, the lack of data from countries outside North America, Europe, Asia and Oceania limit the generalizability of these findings to other countries with different climates, urban environments, socio-political cultures and resources. Despite these limitations, the estimates for the effects of urban trails on measures of PA and AT provided are the most robust to date. Future studies of natural experiments should avoid the methodological errors identified here to enhance the causal estimate of the effect of urban trails on PA, AT behaviours and cycling traffic.

## Conclusions

The following systematic review and meta-analysis provides tentative empirical evidence that the implementation of urban trails increases levels of PA among individuals living in proximity to them. Data also suggest that new urban trails may increase cycling traffic and possibly rates of AT/cycling trips, however larger studies are needed to confirm these estimates. Finally, there is a need for future studies to use more robust epidemiological designs that address assumptions for causal inference and consider measures of equity and justice in research designs to determine if new trails are effective for increasing PA and AT for all urban residents.

## Electronic supplementary material

Below is the link to the electronic supplementary material.

Supplementary Material 1

## Data Availability

No datasets were generated or analysed during the current study.
