# Supplementary material for: The effectiveness of new urban trail infrastructure on physical activity and active transportation: a systematic review and meta-analysis of natural experiments
Source: Int J Behav Nutr Phys Act. 2025 Mar 27;22:36. doi: 10.1186/s12966-025-01729-4 (PMC11951833; doi:10.1186/s12966-025-01729-4)
Supplement: Supplementary file 1 — Supplementary Material 1 [file 12966_2025_1729_MOESM1_ESM.docx]

**Table 1: Search Strategies and Results**

**MEDLINE**

| bicycling/ and (transportation/ or exp environment design/ or cities/ or urban renewal/ or urban population/ or urban health/) | 1355 |
| --- | --- |
| ((bicycle* or bicyclist* or bicycling or bike* or biking or cyclist* or cycling or active transport* or active commut* or active travel*) adj3 (separat* or segregat* or trail* or lane* or path or paths or pathway* or infrastructure* or network* or dedicat* or route* or boulevard* or highway* or built environment* or environment* design or friendl* or sidewalk* or walkway*)).ti,ab,kf. | 1634 |
| ((multiuse or multi use* or shared use* or mixed use* or segregat* or divided or protected or offroad* or off road*) adj2 (path or paths or pathway* or trail* or lane* or route* or sidewalk* or walkway*)).ti,ab,kf. | 770 |
| (urban trail* or greenway or greenways or bikeab* or bikab* or bridleway* or sidepath* or bikeway* or cycleway* or bike way* or cycle way* or railtrail* or rail trail* or foreshoreway* or oceanway* or neighbo?rway* or byway*).ti,ab,kf. | 278 |
| or/1-4 | 3582 |
| comparative study.pt. | 1912800 |
| **e**xp epidemiologic studies/ or control groups/ | 3154086 |
| ((case adj3 (control* or compar* or compeer* or refer* or base*)) or ((control* or intervention*) adj group*) or synthetic control* or cohort* or ((quasi or natural) adj2 experiment*) or quasiexperiment* or time series or before-after or "before and after" or "difference in difference" or longitudinal or ((epidemiologic* or followup or follow up or controlled or comparative or comparison) adj2 (studies or study)) or pretest post* or prepost* or pre-post* or pre test post* or preintervention* or pre intervention* or preimplement* or pre implement*).ti,ab,kf. | 2563022 |
| or/6-8 | 6109766 |
| 5 and 9 | 932 |
| limit 10 to (english language and yr="2010 -Current") | 742 |

**Embase**

| (cyclist/ or cycling/ or bicycle/) and ("traffic and transport"/ or exp environmental planning/ or city/ or exp urban area/ or urban health/ or urban population/ or exp city planning/) | 1602 |
| --- | --- |
| ((bicycle* or bicyclist* or bicycling or bike* or biking or cyclist* or cycling or active transport* or active commut* or active travel*) adj3 (separat* or segregat* or trail* or lane* or path or paths or pathway* or infrastructure* or network* or dedicat* or route* or boulevard* or highway* or built environment* or environment* design or friendl* or sidewalk* or walkway*)).ti,ab,kw. | 1873 |
| ((multiuse or multi use* or shared use* or mixed use* or segregat* or divided or protected or offroad* or off road*) adj2 (path or paths or pathway* or trail* or lane* or route* or sidewalk* or walkway*)).ti,ab,kw. | 945 |
| (urban trail* or greenway or greenways or bikeab* or bikab* or bridleway* or sidepath* or bikeway* or cycleway* or bike way* or cycle way* or railtrail* or rail trail* or foreshoreway* or oceanway* or neighbo?rway* or byway*).ti,ab,kw. | 305 |
| or/1-4 | 4246 |
| comparative study/ or quasi experimental study/ | 1026341 |
| control group/ or pretest posttest control group design/ or pretest posttest design/ or case control study/ or population based case control study/ or static group comparison/ or cohort analysis/ or time series analysis/ or exp longitudinal study/ or follow up/ | 3237283 |
| ((case adj3 (control* or compar* or compeer* or refer* or base*)) or ((control* or intervention*) adj group*) or synthetic control* or cohort* or ((quasi or natural) adj2 experiment*) or quasiexperiment* or time series or before-after or "before and after" or "difference in difference" or longitudinal or ((epidemiologic* or followup or follow up or controlled or comparative or comparison) adj2 (studies or study)) or pretest post* or prepost* or pre-post* or pre test post* or preintervention* or pre intervention* or preimplement* or pre implement*).ti,ab,kw. | 3712297 |
| or/6-8 | 6159777 |
| 5 and 9 | 719 |
| limit 10 to (english language and yr="2010 -Current") | 615 |

**CINAHL**

| ((MH bicycles) or (MH cycling)) and ((MH transportation) or (MH "built environment") or (MH "universal design") or (MH "urban areas") or (MH "urban population") or (MH "urban health/")) | 606 |
| --- | --- |
| ((bicycle* or bicyclist* or bicycling or bike* or biking or cyclist* or cycling or (active N1 transport*) or (active N1 commut*) or (active N1 travel*)) N3 (separat* or segregat* or trail* or lane* or path or paths or pathway* or infrastructure* or network* or dedicat* or route* or boulevard* or highway* or (built N1 environment*) or (environment* N1 design) or friendl* or sidewalk* or walkway*)) | 497 |
| ((multiuse or ((multi or shared or mixed) N1 use*) or segregat* or divided or protected or offroad* or (off N1 road*)) N2 (path or paths or pathway* or trail* or lane* or route* or sidewalk* or walkway*)) | 135 |
| ((urban N1 trail*) or greenway or greenways or bikeab* or bikab* or bridleway* or sidepath* or bikeway* or cycleway* or (bike N1 way*) or (cycle N1 way*) or railtrail* or (rail N1 trail*) or foreshoreway* or oceanway* or neighbo#rway* or byway*) | 401 |
| S1 or S2 or S3 or S4 | 1480 |
| (MH "comparative studies") or (MH "quasi-experimental studies+") or (MH "case control studies+") or (MH "pretest-posttest design+") or (MH "controlled before-after studies") or (MH "historically controlled study") or (MH "interrupted time series analysis") or (MH "prospective studies+") or (MH "control group") | 1010324 |
| ((case N3 (control* or compar* or compeer* or refer* or base*)) or ((control* or intervention*) N1 group*) or (synthetic N1 control*) or cohort* or ((quasi or natural) N2 experiment*) or quasiexperiment* or "time series" or "before-after" or "before and after" or "difference in difference" or longitudinal or ((epidemiologic* or followup or "follow up" or controlled or comparative or comparison) N2 (studies or study)) or (pretest N1 post*) or prepost* or (pre N1 post*) or ("pre test" N1 post*) or preintervention* or (pre N1 intervention*) or preimplement* or (pre N1 implement*)) | 1192756 |
| S6 or S7 | 1421601 |
| S5 and S8 | 424 |
| S9 Limiters - Published Date: 20100101-; English Language | 348 |

**SPORTDiscus**

| (ZE "bicycle facilities") or (ZE "bicycle trails") or (ZE "urban cycling") or (ZE "bicycle commuting") | 1615 |
| --- | --- |
| ((bicycle* or bicyclist* or bicycling or bike* or biking or cyclist* or cycling or (active N1 transport*) or (active N1 commut*) or (active N1 travel*)) N3 (separat* or segregat* or trail* or lane* or path or paths or pathway* or infrastructure* or network* or dedicat* or route* or boulevard* or highway* or (built N1 environment*) or (environment* N1 design) or friendl* or sidewalk* or walkway*)) | 6574 |
| ((multiuse or ((multi or shared or mixed) N1 use*) or segregat* or divided or protected or offroad* or (off N1 road*)) N2 (path or paths or pathway* or trail* or lane* or route* or sidewalk* or walkway*)) | 207 |
| ((urban N1 trail*) or greenway or greenways or bikeab* or bikab* or bridleway* or sidepath* or bikeway* or cycleway* or (bike N1 way*) or (cycle N1 way*) or railtrail* or (rail N1 trail*) or foreshoreway* or oceanway* or neighbo#rway* or byway*) | 766 |
| S1 or S2 or S3 or S4 | 7430 |
| ((case N3 (control* or compar* or compeer* or refer* or base*)) or ((control* or intervention*) N1 group*) or (synthetic N1 control*) or cohort* or ((quasi or natural) N2 experiment*) or quasiexperiment* or "time series" or "before-after" or "before and after" or "difference in difference" or longitudinal or ((epidemiologic* or followup or "follow up" or controlled or comparative or comparison) N2 (studies or study)) or (pretest N1 post*) or prepost* or (pre N1 post*) or ("pre test" N1 post*) or preintervention* or (pre N1 intervention*) or preimplement* or (pre N1 implement*)) | 171534 |
| S5 and S6 | 154 |
| S7 Limiters - Published Date: 20100101-; English Language | 108 |

**Web of Science Core Collection**

| TS=((bicycle* or bicyclist* or bicycling or bike* or biking or cyclist* or cycling or (active NEAR/1 transport*) or (active NEAR/1 commut*) or (active NEAR/1 travel*)) NEAR/3 (separat* or segregat* or trail* or lane* or path or paths or pathway* or infrastructure* or network* or dedicat* or route* or boulevard* or highway* or (built NEAR/1 environment*) or (environment* NEAR/1 design) or friendl* or sidewalk* or walkway*)) | 30852 |
| --- | --- |
| TS=((multiuse or ((multi or mixed or shared) NEAR/1 use*) or segregat* or divided or protected or offroad* or (off NEAR/1 road*)) NEAR/2 (path or paths or pathway* or trail* or lane* or route* or sidewalk* or walkway*)) | 5899 |
| TS=((urban NEAR/1 trail*) or greenway or greenways or bikeab* or bikab* or bridleway* or sidepath* or bikeway* or cycleway* or (bike NEAR/1 way*) or (cycle NEAR/1 way*) or railtrail* or (rail NEAR/1 trail*) or foreshoreway* or oceanway* or neighbo$rway* or byway*) | 2383 |
| #1 or #2 or #3 | 38520 |
| TS=((case NEAR/3 (control* or compar* or compeer* or refer* or base*)) or ((control* or intervention*) NEAR/1 group*) or (synthetic NEAR/1 control*) or cohort* or ((quasi or natural) NEAR/2 experiment*) or quasiexperiment* or "time series" or "before-after" or "before and after" or "difference in difference" or longitudinal or ((epidemiologic* or followup or "follow up" or controlled or comparative or comparison) NEAR/2 (studies or study)) or (pretest NEAR/1 post*) or prepost* or (pre NEAR/1 post*) or ("pre test" NEAR/1 post*) or preintervention* or (pre NEAR/1 intervention*) or preimplement* or (pre NEAR/1 implement*)) | 3917505 |
| #4 and #5 | 2337 |
| #6 Timespan: 2010-01-01 to 2023-12-31 (Publication Date) | 1981 |
| Refined by: Languages: English | 1958 |

**TRID**

| Index terms:bicycle commuting or bicycle travel or bicycle lanes or bikeways or greenways Language:English | 3947 |
| --- | --- |
| index terms:city planning and bicycling Language:English | 113 |
| index terms:city planning and cyclists Language:English | 108 |
| keywords:((bicycle* or bicyclist* or bicycling or bike* or biking or cyclist* or cycling or "active transport" or "Active transportation" or "active commuter" or "active commuting" or "active commute" or "active travel") AND (separat* or segregat* or trail* or lane* or path or paths or pathway* or infrastructure* or network* or dedicat* or route* or boulevard* or highway* or "built environment" or friendl*)) Language:English | 89 |
| keywords:("urban trail" or greenway or greenways or bikeab* or bikab* or bridleway* or sidepath* or bikeway* or cycleway* or "bike way" or "cycle way" or railtrail* or "rail trail" or foreshoreway* or oceanway* or neighborway* or neighbourway" or byway*) Language:English | 21 |
| 1 or 2 or 3 or 4 or 5 | 4112 |
| keywords:("case control" or "case controlled" or "case compare" or "case comparison" or "case compeer" or "case referent" or "case referrent" or "case based") | 867 |
| keywords:("longitudinal study") | 371 |
| keywords:("epidemiologic study" or "epidemiological study") | 101 |
| keywords:("followup study" or "follow up study" or "controlled study" or "comparative study" or "comparison study") | 4047 |
| keywords:("control group" or "control groups" or "intervention group" or "intervention groups" or "synthetic control") | 1766 |
| keywords:("quasi experimental" or "natural experiment" or quasiexperiment*) | 485 |
| keywords:("time series" or "before-after study" or "before and after study") | 6205 |
| keywords:("pretest posttest" or prepost* or "pre post" or "pre posttest" or "pre test post" or "pre test post test" or preintervention* or "pre intervention" or preimplement* or "pre implementation") | 352 |
| 7 or 8 or 9 or 10 or 11 or 12 or 13 or 14 | 13725 |
| 6 and 15 | 92 |
| limit to 2010-present | 67 |

**Google Scholar**

| bicycle\|cyclist\|bicycling\|cycling\|bike\|biking separated\|trail\|lane\|path\|pathway\|infrastructure\|network\|bikeability\|greenway\|bikeway\|cycleway control\|controlled\|comparative\|compared\|comparison\|longitudinal\|"natural experiment"\|quasiexperimental\|"time series"\|"before after"\|pretest since 2010, first 10 pages | 98 |
| --- | --- |

**Table 2: Risk of Bias**

**Figure s1.** Sensitivity analysis of changes in PA between individuals living in intervention and control areas restricted to those within the closest geographic buffer.

**
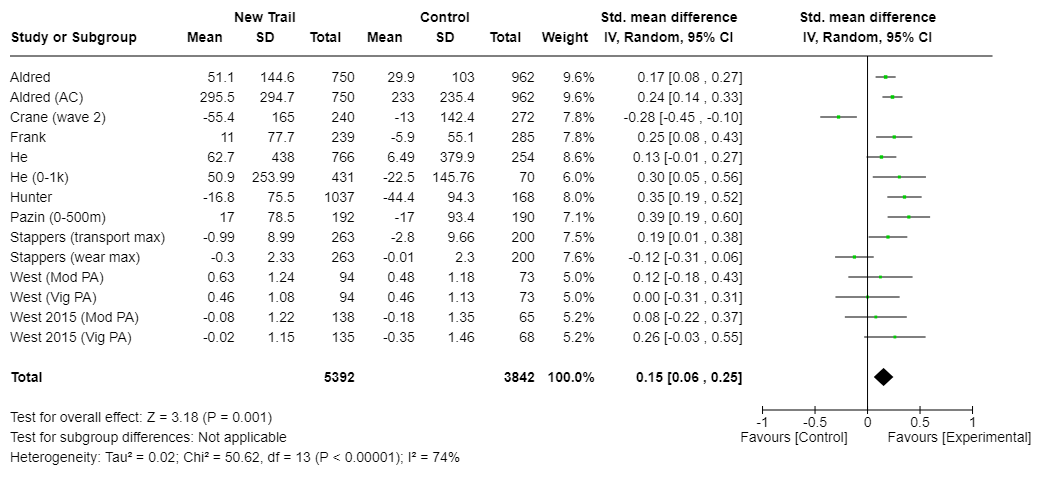
**
